# Adolescents' experiences of being food-hypersensitive: a qualitative study

**DOI:** 10.1186/1472-6955-6-8

**Published:** 2007-10-09

**Authors:** Birgitta Marklund, Bodil Wilde-Larsson, Staffan Ahlstedt, Gun Nordström

**Affiliations:** 1 Centre for Allergy Research, Karolinska Institutet, S-171 77 Solna, Sweden; 2 Department of Neurobiology, Care Sciences and Society, Division of Nursing, 23300, Karolinska Institutet, S-141 83 Huddinge, Sweden; 3 Department of Nursing, Karlstad Universitet, S-651 88 Karlstad, Sweden; 4 National Institute of Environmental Medicine, Karolinska Institutet, S-171 77 Solna, Sweden

## Abstract

**Background:**

Experiencing or being at risk of adverse reactions to certain food items is a common health issue, especially among children and adolescents. Research has shown that living with the risk of food reactions and always having to take measures to avoid certain food in one's diet has a negative impact on quality of life. The aim of this study was to illuminate adolescents' experiences of being food hypersensitive.

**Methods:**

Three focus group interviews and six individual interviews were carried out with all together 17 adolescents, 14–18 years of age, who had exclusion diets at school due to food hypersensitivity. The interviews were taped and transcribed verbatim and a qualitative content analysis was carried out.

**Results:**

Five categories with subcategories, and one pervading theme, emerged. The categories were: *Perceiving oneself as being particular*, *Feeling constrained*, *Experiencing others' ignorance*, *Keeping control*, and *Feeling it's okay*. A pervading theme was conceptualised as *Striving to normalise the experience of being food-hypersensitive*. The adolescents regarded themselves as competent and courageous, but also described how they avoided the extra attention it implied to ask for special food considerations taken into account. Their self-conceptions were probably essential for their management of and attitude toward the hypersensitivity condition. They felt deprived, and those at risk of severe food reactions experienced insecurity and fear. Feelings of being disregarded were expressed, as well as facing unreliability and a lack of understanding from others. The continual work of constant vigilance and decision-making was described as time-consuming and frustrating. However, the adolescents also experienced considerate and supportive surroundings and were at pains to tone down the negative experiences and consequences of being food-hypersensitive.

**Conclusion:**

Food avoidance by itself, and not only the somatic food reactions, brings about consequences with significant impacts on adolescents' lives. The findings from this study have implications for all of those who deal with adolescents who are food-hypersensitive, and not only health professionals. A deeper insight into adolescents' experiences gives an understanding which can improve the care-givers' efforts.

## Background

Experiencing or being at risk of adverse reactions to certain food items is a common health issue, especially among children and adolescents. The prevalence of food allergy is 1–2%, and approximately another 20% avoid certain foods due to other kinds of food hypersensitivity problems [1]. According to the European Academy of Allergology and Clinical Immunology nomenclature for allergy [2], the term "food hypersensitivity" is used for both allergic and non-allergic hypersensitivity. This term is also used in the present study as an overall concept regardless of the underlying mechanisms of the adverse food reactions, also including food intolerance such as gluten intolerance and lactose intolerance.

Research has shown that food hypersensitivity is a condition with a significant impact on quality of life, both in children and adults [3-5]. Family and social life is negatively affected [4,6] and parents report that food-hypersensitive children are limited in their autonomous social activities [7,8]. Many children can never sleepover at a friend's house or join camp school due to their food hypersensitivity [7] and adolescents have a higher absence from school compared to controls [9]. Children with peanut allergy experience more anxiety about eating than children with diabetes mellitus [10] and they have more daily disruptions and family social impairment than children with rheumatological disease [11]. Food hypersensitivity has a negative impact on family economy [7] and participation in the work force [12]. Moreover, food-hypersensitive adolescents report less self-confidence [9], and parents report worries, health and nutritional concerns, and frustration over time-consuming precautionary measures [6].

The most common clinical expressions of food hypersensitivity are symptoms from the gastrointestinal tract, followed by cutaneous or respiratory symptoms and systemic anaphylaxis [13]. A US study has shown that adolescents as a group are most at risk of fatal food reactions, often in settings outside the home and school [14]. Consistent and successful avoidance of the unsafe foods, however, limits the influence of food hypersensitivity on the individual's physical health. Yet, living with the risk of food reactions and always having to take measures to avoid certain food in one's diet has a negative impact on quality of life [15].

Adolescence is a period of development that affects the adolescents' way of managing psychological and social issues [16]. Our knowledge today about what impact food hypersensitivity has on children's and adolescents' quality of life is mainly based on parents' reports [3,4,6-8,17]. Research on food hypersensitivity seen from the adolescents' perspective is currently lacking. In order to better understand and support food-hypersensitive adolescents and perhaps facilitate avoidance of unsafe foods, there is a need for more in-depth knowledge on how the adolescents experience being food-hypersensitive. Thus, the aim of this study was to illuminate adolescents' experiences of being food-hypersensitive.

## Methods

### Informants

Interviews were carried out with 17 adolescents who had exclusion diets at school due to food hypersensitivity. They were all pupils at the senior level of the nine-year compulsory school and at the upper secondary school in a municipality in the southern region of Stockholm County, Sweden. The adolescents were recruited from the sample of a previous study on children and adolescents with exclusion diets at school due to food hypersensitivity [18]. Thus, they were considered to be at risk of adverse reactions but they were not necessarily patients at a medical clinic. From this sample all adolescents, 13–19 years of age, were invited to participate in sex-specific focus group interviews or, if they declined focus group participation, in individual interviews about their experiences of being food-hypersensitive. There were 41 eligible adolescents in these age groups who were offered participation, of which 17 (10 females and 7 males, aged 14–18 years) agreed to be interviewed, 11 in focus groups and six individually.

### Interviews

All interviews were conducted by one of the authors (BM) between November 2002 and April 2003. The focus-group interviews (two female groups and one male group with a total of 11 participants) took place at the County Hall with an observer present. The observer (registered nurse, psychologist and doctoral student in allergy research) was attentive to non-verbal communication and interaction between participants. After each focus-group interview the interviewer and the observer shared their perceptions of the group interaction and its significance for the data analysis. The individual interviews took place in the adolescents' homes (n = 1), at school (n = 1) or at the County Hall (n = 4), in accordance with each adolescent's preference. Both the focus groups and the individual interviews started with a question about the adolescents' hypersensitivity characteristics (offending food items, symptoms and onset), which are summarised in Table 1. Afterwards, an open question on how the adolescents' experienced being food-hypersensitive was put, followed by individually adapted questions covering experiences from school and leisure time, family life and health care. The adolescents were encouraged to speak freely about their thoughts, feelings, and actions about these matters, and to elaborate on their responses throughout the interviews. The individual interviews lasted 30–90 minutes and the focus-group interviews 90–120 minutes. All interviews were audio-taped and transcribed verbatim by BM.

**Table 1:** Characteristics of the interviewed adolescents, 13–18 years, with food hypersensitivity (self-reported data).

	**Individual (I) or group (G) interview**	**Offending foods**	**Adverse food reaction(s)**	**Onset**	**Positive diagnostic test(s)**	**Additional family member(s) with food hyper-sensitivity**
Boy 15 years	G1	Fish, nuts	Breathing difficulties, conjunctivitis	Early childhood	Yes	No
Boy 16 years	G1	Vegetables, fruits, nuts	Breathing difficulties, itchy face, rash	Early childhood	Yes	Yes
Boy 15 years	G1	Fish, nuts, egg, gluten	Itching	Early childhood	Yes	Unknown
Girl 15 years	G2	Orange	Eczema	Age 8 y	Yes	No
Girl 14 years	G2	Tomato	Breathing difficulties, rash	Age 9 y	Yes	Yes
Girl 15 years	G2	Gluten		Age 1,5 y	Yes	No
Girl 14 years	G2	Egg, lactose	Rash, itchy throat	Age 9 y	Yes	Yes
Girl 18 years	G3	Citrus, gluten	Eczema, mood swings	Early childhood	Yes	No
Girl 16 years	G3	Shellfish	Rash, itchy/swollen mouth and throat	Age 15 y	No	No
Girl 17 years	G3	Fruits, nuts, beans, peas	Breathing difficulties	Early childhood	Yes	Unknown
Girl 16 years	G3	Fruits, almonds, paprika	Asthma, rash	Age 6 y	Yes	Yes
Girl 17 years	I	Egg, nuts, fruits	Gastro-intestinal pain, itchy/swollen mouth and throut, vomiting	Early childhood	Yes	Yes
Boy 14 years	I	Wheat, peas, nuts, soy, tomato	Gastro-intestinal pain, itchy/swollen mouth and throat, diarrhoea	Early childhood	Yes	Yes
Boy 17 years	I	Fruits, peanuts, soy	Itchy/swollen Mouth and throat	Early childhood	Yes	No
Boy 16 years	I	Tomato	Itchy/swollen mouth and throat	Unknown	Yes	Yes
Boy 14 years	I	Tomato, nuts, rosehip, lactose	Gastro-intestinal pain, vomiting, rash	Early childhood	Yes	Yes
Girl 18 years	I	Tomato, fish, shellfish	Breathing difficulties, gastro-intestinal pain, eczema	Early childhood	No	Yes

### Data analysis

To get a first overall sense of the interview content and to validate the transcripts, the texts were read through and the tapes were listened to simultaneously. Qualitative content analysis was carried out using the procedures laid out by Graneheim and Lundman [19]. Qualitative content analysis is often used in nursing and education, and interpretation of the content is an essential part of the data analysis. This approach differs from quantitative content analysis, which deals with quantitative descriptions of the manifest content of the data [19]. All the transcripts were analysed in similar ways, regardless of focus-group or individual interview.

Each transcript was read line by line and the adolescents' descriptions of actions, thoughts and feelings with regards to the experience of being food-hypersensitive were identified and given names, i.e. codes. Codes with similar meanings were grouped together in categories. As the analysis process proceeded, categories and subcategories were subsequently clarified and adjusted.

The concluding categories and subcategories (Table 2) constitute the manifest content of the text. One pervading theme was identified, constituting the underlying meaning or the latent content of the text [19]. The initial coding of the transcripts was performed by BM, and the coded data were examined by BM, GN and BW for emergent theme, categories and subcategories. The interpretations were compared and discussed until consensus was reached.

**Table 2:** Theme, categories and subcategories that emerged from interviews with adolescents concerning their experiences of being food-hypersensitive.

**Theme**	**Striving to normalise the experience of being food-hypersensitive**				
**Categories**	**Perceiving oneself as being particular**	**Feeling constrained**	**Experiencing others' ignorance**	**Keeping control**	**Feeling it's okay**

**Subcategories**	The body is insufficient	Deprivation	Disregard	Vigilance	Personal competence
	Psychological and emotional impact	Insecurity	Unreliability	Tests and evaluations	Considerate and supportive surroundings
	Being a bother	Fear of severe food reactions	Lack of understanding	Making decisions	It could be worse

### Ethical considerations

All adolescents and their parents were informed by letter of the objective and design of the study and told that the participants were free to leave the interview if they wished. Oral consent was received from all participants and from a parent of each adolescent. The study was approved by the Research Ethics Committee at Huddinge University Hospital, Sweden.

## Results

From the transcribed interviews five categories with subcategories, and one pervading theme, emerged. The categories were: *Perceiving oneself as being particular*, *Feeling constrained*, *Experiencing others' ignorance*, *Keeping control*, and *Feeling it's okay*. A pervading theme was conceptualised as *Striving to normalise the experience of being food-hypersensitive *(Table 2).

### Perceiving oneself as being particular

The category *Perceiving oneself as being particular*, in the sense of being special or different, describes the perception of self, i.e. aspects of the biopsychosocial identity of the food-hypersensitive adolescent, as they emerged from the interviews. Three subcategories were identified: *The body is insufficient *(bio), *Psychological and emotional impact *(psycho), and *Being a bother *(social). Although the adolescents expressed that there was "something wrong" with their body, they did not consider themselves as having a disease. Furthermore, though the adolescents expressed feelings of being outsiders, they also felt that the condition had become a constructive part of their identity. In their relations to others they could perceive themselves as being a bother.

The way the adolescents described themselves as food-hypersensitive individuals, together with their thoughts on physiological food hypersensitivity mechanisms, revealed that they made a distinction between themselves and "normal persons".

#### The body is insufficient

The adolescents believed that the food-hypersensitive body was insufficient. Something was missing, or there was a deformity, often explained as being genetic. Words like "defect", "short-circuit", "misinterpretation" and "mistake" were used to explain the insufficiency. Moreover, those who required nutritional supplements such as extra vitamins or minerals because of food avoidance due to hypersensitivity, seemed to consider this a sign of body insufficiency.

Despite the perception of an insufficient body, and despite the fact that the adolescents were aware of the limitations the condition sometimes implied, they did not consider their food hypersensitivity to be a disease. One girl said:*"Everybody is different. This is my little kind of difference, sort of."*

#### Psychological and emotional impact

The adolescents described both positive and negative psychological and emotional impacts of being food-hypersensitive. It was emphasised that the condition required both courage and a strong will, and that it required self-confidence to ask for alternative foods without feeling uncomfortable.

Food hypersensitivity led to experiences and consequences that made them develop in a positive way. In some way the hypersensitivity had become a part of their identity. For example, one girl stated that being food-hypersensitive had given her increased empathic skills.

"I have heard from my friends, my closest friends, they say that they often talk to me about problems and things. They think that I understand somehow, listen and understand and so on. Perhaps I would not have done that quite as well (...) I have several times thought that if I could, I would conjure away my allergy – but in a way it feels like it wouldn't be me anymore. Then it would be a part of me that disappears."

However, being food-hypersensitive could also imply psychologically destructive experiences. There were examples of adolescents who had been subjected to teasing and even harassment, and felt that this was related to the fact that they were food-hypersensitive.

The extra attention that food hypersensitivity involved could be experienced as something agreeable. However, feelings of being an outsider or getting in awkward situations were also expressed. For example, the routines of the school-meal services led to a separation from "normal persons" with unwanted and unpleasant attention as a consequence.

"Well, when it was tomato soup or whatever, normal persons got their food and those who were hypersensitive to it had to go through another entrance via the kitchen and get it directly from the storeroom. (...) You felt somehow special in a way and that wasn't something you wanted. Didn't want to stand out like that."

Emotions like sadness and anger at their condition, envy of non-hypersensitive friends and a wish to be like everybody else were also expressed.

#### Being a bother

Besides the embarrassment of the extra attention it sometimes involved in asking for non-offending food, the adolescents described the feeling of being a bother and causing trouble to others. They perceived that their special requests concerning foods involved extra work for their friends.

"I think it is difficult because they might say 'well then, but what shall we cook then?' Then you feel like this, that you are a hassle to those who do the cooking. I feel like, 'well then, now I'm a bother again'."

### Feeling constrained

Besides the hard work of keeping control of the food intake, the adolescents described constraining effects of their food hypersensitivity. Limitations in possible food and eating-places had negative practical and social consequences. Their food hypersensitivity condition could in new and unknown situations bring about a sense of insecurity. Having to abstain from certain foods, but also feeling insecure, were some of the constraints described by the adolescents. A crucial part of the food hypersensitivity experience was being at risk of, and fearing, adverse reactions. These kinds of experiences and feelings were a burden on the adolescents, and are included in the category *Feeling constrained*. There are, thus, three subcategories: *Deprivation*, *Insecurity*, and *Fear of severe food reactions*.

#### Deprivation

The food-hypersensitive adolescents experienced deprivation in many ways. Some expressed sadness about having to abstain from certain foods and not being able to eat some favourite dishes. Limited choices of restaurants and restrictions in attending certain parties such as crayfish parties affected their social life negatively.

Although the adolescents might say that the elimination diet served at school tasted good, there were complaints about the food quality, which they thought was not as good as for the "ordinary" food. Furthermore, the routines of the school-meal services sometimes led the food-hypersensitive adolescent to having to leave the general queue for food and fetch her/his food from another place. This was felt by the adolescents to be a negative discriminative procedure.

"It was quite hard in the beginning. When you were standing there in a queue, joking and talking, you had to go into another bloody room to get your food. So then, when you came out again everybody was already seated and it was full at the table and you ended up sitting on your own. It sort of created ... there were a lot of chain reactions as a result of this."

#### Insecurity

The only place where the adolescents felt completely safe with regards to unsafe foods was at home. Being away from home or in unknown environments caused a sense of insecurity and involved extra precautions. The degree of insecurity among adolescents at risk of severe reactions was increased with the distance to everyday life, for example when starting at a new school or when on holiday abroad.

"I do swimming a lot, and there are many camps and you are away from home. If you are abroad or far away from a hospital, you get a little worried and are more concerned about what you eat and so on. In anyway if it's a long way to go to a hospital."

#### Fear of severe food reactions

Severe adverse reactions, such as food-induced breathing difficulties, caused fear. Knowing from previous reactions that breathing difficulties involved anxiety and anguish, and knowing that a systemic reaction could be lethal, was frightening, not only at the time of the reaction but also after such an adverse reaction. During periods of time after a severe food reaction, the adolescents' vigilance increased. *"Now, I don't dare to eat anything with fish or tomato because I'm scared it might happen again."*

The adolescents also experienced fear when they had mistakenly eaten something dangerous such as nuts, or discovered that the eaten food item could contain traces of nuts. Merely the smell of nuts could involve fear of reactions.

"I react very strongly to nuts, all kind of nuts. If you put some nuts on a table, that is all that is needed for me not to be able to be in that room. So, when you eat cereals and so on, and it says on the package that it can contain traces of nuts... then .. uuuh, 'just a second, what have I eaten?' (...) You sure get a little bit frightened."

Some adolescents at risk of severe reactions carried self-injectable epinephrine with them. This is important medical equipment in case of an acute systemic reaction to an offending food. To carry such an "adrenaline pen" could moderate the fear, but there was still a fear of having to use the medicine. *"I also do worry about that sometimes (...) that I have to take that disgusting injection."*

### Experiencing others' ignorance

The interviewed adolescents experienced that people around them had little knowledge about food hypersensitivity and sometimes that their special needs with regards to food were not fully understood. The adolescents were aware of the fact that non-hypersensitive individuals might not have a complete understanding of what it meant to be food-hypersensitive. However, the adolescents described several occasions when their special needs had been disregarded, when the environment was experienced as unreliable, and when they were frustrated over the lack of understanding from others. It was mostly adults, and not those of the same age, who showed such ignorance of the importance of complying with the adolescents' special food needs.

The ignorance of others seemed to be a major obstacle for the adolescents' in their efforts to keep control of their food intake. The category *Experiencing others' ignorance *involves three subcategories: *Disregard*, *Unreliability*, and *Lack of understanding*.

#### Disregard

The adolescents might have no problems in understanding or making excuses for, for example older relatives who unintentionally forgot that they could not eat certain foods.

"For example, when you are at your grandma's or something like that. They have bought cookies and stuff, and there are nuts in everything. They feel a little bad about having forgotten the problem."

However, the adolescents commented adversely on domestic science teachers' and school-meal service personnel's negligence to plan for the food-hypersensitive adolescents' special needs at school. For example, it could happen that the central kitchen forgot to prepare the elimination diet for a certain adolescent. Also on school excursions and at sport camps the adults sometimes forgot to plan for the food-allergic adolescents' exclusion diets. On such occasions, the adolescents felt disregarded. *"Well, it felt like this, 'okay, you didn't think about me"'*.

The school cafeteria did not offer gluten-free sandwiches despite requests. This was interpreted as an unwillingness to show consideration for such a small group that the gluten-intolerant pupils constituted.

#### Unreliability

The adolescents gave several examples of experiencing unreliable environments. The information from restaurant and school-meal service personnel, friends and relatives could be incomplete and even incorrect, sometimes because of inadequate ingredient labels.

"There have been several times when I have asked if there were eggs in the food, and they thought it over and said 'No, there aren't'. So I have eaten it, and then I got sick afterwards because there were eggs in it."

The ingredients in foodstuffs in stores or in dishes served at school or a restaurant could be changed without further information. Furthermore, the peanut-allergic adolescents could never be sure that they were not being exposed to peanuts in public places.

"Some weeks ago, we were at the theatre with the school and they sold peanuts during the interval. And then people started to open the peanut bags in the auditorium and it started to itch in my throat and my mouth. (...) It is frustrating and annoying. I think it is pretty bad that they sell peanuts at all at theatres."

##### Lack of understanding

The adolescents experienced that other people did not understand the seriousness of their condition.

"Some, especially adults, they don't take it seriously when I tell them that I'm peanut-allergic (...) they think you exaggerate. In fact, it is mostly adults. When you are at a restaurant and so on. So you have to make it clear to them that if I eat this it ... it'll be the ambulance that's next."

It also happened that restaurant personnel gave the impression of indifference to their requests for special meal orders. There were also complaints from the adolescents about the bad quality of the exclusion diet served at school, which they thought was a result of the personnel's lack of knowledge and lack of interest in learning how to cook tasty elimination diet meals.

*"When I went to the senior level (of the nine-year compulsory school), my mother volunteered to come to school and teach them. But they didn't want her to, because they were a bit arrogant and thought that 'of course we know this'"*.

### Keeping control

An important task in managing food hypersensitivity was to keep control of the food intake. This was done by inspecting the served food, asking what the food contained, reading labels and menus, and sometimes testing to see if the food was okay to eat. With the help of allergy tests and their own evaluation of possible reactions, the adolescents could judge and decide whether the food was safe to eat or not. The category *Keeping control *involves three subcategories: *Vigilance*, *Tests and evaluations*, and *Making decisions*.

#### Vigilance

In order to manage their food hypersensitivity, the adolescents' always had to check for unsafe foods in all situations involving food, i.e. they had to be vigilant. When away from home they inspected the served food for unsafe food items or asked what the food contained. At restaurants they read the menus carefully and also asked the personnel what the food contained. In food stores they read the ingredient labels. They sometimes described this a responsibility that was obvious and not considered to be a problem. *"I usually ask. It comes automatically."*

However, although it was an indisputable matter of course to always check for unsafe foods, it was also described as troublesome and hard work. The task was experienced as being a demanding part of food-hypersensitivity management and was described as bothersome, frustrating, time-consuming, and sometimes embarrassing.

In stressful situations watchfulness could be reduced, with food reactions as a consequence. This could sometimes lead to self-reproach. It was emphasised that it was the adolescents' own responsibility to make sure that the food was safe to eat. *"It is solely my responsibility to check it up. If I don't, I only have myself to blame."*

#### Tests and evaluations

Adolescents with multiple and/or severe food allergies appreciated regular allergy tests at an allergy clinic. One boy with no health-care contacts wished to establish such a contact in order to get about a better understanding of his hypersensitivity condition. Furthermore, the tests were seen as an aid to identifying the offending foods, and also to getting a picture of how their hypersensitivity changed over time.

The adolescents said that they knew, not only from the tests, but also from their own experiences what they could eat and what they had to avoid. To evaluate possible changes in reactions was described as one way of testing and evaluating their hypersensitivity.

"It was one or two months ago I tested to eat a boiled egg, just to see if I could, although test results have shown a 'five'. But nothing happened. (...) If I can eat a boiled egg, it should be possible for me to eat food that contains egg."

#### Making decisions

In all situations involving food the adolescents had to estimate the risks and decide whether to eat the food or not, or to ask for alternative foods. Especially on holiday trips, camps and other occasions when away from home, the management of the meals required extra reflection and planning.

"When we have arrived and unpacked, we find out where there are restaurants. And then we compare them and so on. Which ones have the best food for me (...) and we compare so we can decide which restaurant can be okay for all the family."

If they were insecure about the ingredients, the adolescents chose to abstain from eating, especially when there were risks of severe adverse reactions. However, some adolescents said that they had a choice. Sometimes a special dish or a special occasion could be worth the pain or discomfort caused by the unsafe food.

"Once I had a (...) pizza. I guess I thought that it was so many years ago since I had eaten one, and it tastes so good so it could be worth some stomach ache. (...) It was worth it. You do have a choice ... always, so you should not feel sorry for yourself."

Also, if it was too embarrassing to ask for alternative foods the adolescent could choose to eat without revealing their food hypersensitivity, despite the risk of adverse reactions.

### Feeling it's okay

From early years the adolescents had been taught by parents and grandparents to avoid certain food. Their food-avoiding skills were developed through label reading but also through increased confidence in asking and making demands. The adolescents felt competent and experienced support from family, friends, school, and health services. A way of making it feel okay to be food-hypersensitive was to relate the condition to other, more difficult problems.

Three subcategories were identified: *Personal competence*, *Considerate and supportive surroundings *and *It could be worse*. Together these three subcategories illustrate aspects of the adolescents' experiences of getting along quite well despite their food hypersensitivity.

#### Personal competence

The adolescents felt competent and secure regarding their own knowledge and ability to judge what foods were safe. *"I can cook, so it's cool. I don't worry at all. I have learnt what to avoid. Read. Check thoroughly."*

Their parents and sometimes grandparents had taught them how to read the labels, what food items they had to avoid, and what alternative foods they could have instead. They felt confident about having a household of their own in the future. In fact, they considered that it would be easier then, when they had to think only about themselves.

#### Considerate and supportive surroundings

When efforts were made at restaurants to provide, for example, lactose- or gluten-free alternatives, this was highly appreciated. So were also friends' and relatives' efforts to comply with the adolescents' special needs concerning food. The adolescents felt totally secure at home, and the system at school for elimination diet supply was for the most part considered to be trustworthy. Pupils with food hypersensitivity usually got their meal from a special table, where all the different foods were labelled, so there should be no doubt about what everyone could eat. These routines made it possible for the adolescents to easily avoid unsafe foods.

Health care services, such as school nurse receptions, allergy clinics, and emergency wards were looked upon as helpful and supportive systems, even though the adolescents complained about the waiting time when visiting an emergency ward because of breathing difficulties. Recurrent health-care contacts were appreciated and it was seen as positive to have contact with a doctor who knew the adolescents' specific hypersensitivity problems and could follow up previous events and check-ups.

"I see a doctor who has been my doctor since I was really small. And he, he somehow knows exactly how I function and how I ... how my allergy behaves and so on. So that feels ... he is really great."

Regular allergy testing in health-care settings was also considered supportive.

#### It could be worse

The adolescents expressed that "it could be worse". They made downward comparisons with for example siblings with more severe hypersensitivity problems, or with themselves in younger years when they had more hypersensitivity problems than now.

"There are people who have things much worse. So you just need to think about them. (...) If I think things are hard for me, they must live through hell every day. I don't even think about it as being hard for me."

There were also attempts to describe the condition as something favourable. *"When other pupils get something disgusting to eat, and I can just eat my pasta with meatballs, then they usually envy me."*

They felt that, compared with some years ago, public knowledge of food hypersensitivity condition and its consequences had increased.

### Striving to normalise the experience of being food-hypersensitive – the pervading theme

One pervading theme, -a thread of underlying meaning that recurred in all categories – emerged: *Striving to normalise the experience of being food-hypersensitive*. The theme expresses the latent content of the text [19].

Although the interviews disclosed negative experiences, critical thoughts and depressive emotions about being food-hypersensitive, there was at the same time a trait of toning down the negative experiences and consequences of being food-hypersensitive. Complaints, critical remarks and negative reports were often followed by extenuating comments. One way for the adolescents to normalise their situation was to look upon food hypersensitivity as something that could happen to anyone.

The adolescents said: *"It is not that bad"*, *"I get along quite well"*, and *"There is no reason to pity me"*. However, it was not always clear if these statements were information directed to the interviewer, or if the adolescents wanted to reassure themselves that it was not all that bad to be food-hypersensitive. The adolescents could shift back and forth between complaints and reassurances that things were all right.

The adolescents commented on the fact that they had grown accustomed to being food-hypersensitive. These comments could have an air of both acceptance and resignation. Although their food hypersensitivity required daily attentiveness, the adolescents expressed that they did not think a lot about their hypersensitivity condition. It seemed to be a deliberate normalising strategy not to dwell on it and, above all, not feel sorry for themselves.

## Discussion

The focus of the present study is on adolescents' experiences of being food-hypersensitive and the theme found to pervade the adolescents' descriptions is conceptualised as *Striving to normalise the experience of being food-hypersensitive*. Although they reported difficulties with and negative experiences of being food-hypersensitive, the adolescents recurrently said that *"it's no big deal" *being food-hypersensitive. This can be seen partly as a copings strategy, and partly as a result of the adolescents getting used to being food-hypersensitive and all that implies.

Feelings of being particular, in the sense of feeling special or different, were revealed by the way the adolescents talked about their body, the impact the condition had had on their personality, and how they felt positioned in their interaction with others. The adolescents described themselves as courageous, but also how they avoided the extra attention it involved asking for special food arrangements. Moreover, 'being particular', in the sense of being selective, touched upon the adolescents' continual work of vigilance in order to avoid certain food. 'Being particular' can be seen as a concept synthesising the biopsychosocial aspects of the food-hypersensitivity identity. One can assume that the adolescents' self-conceptions were shaped by their experiences of living with food hypersensitivity, and their self-conceptions were probably essential for their management of and attitude towards the hypersensitivity condition. Also adolescents with diabetes and adults with gluten intolerance have feelings of being different [20,21]. Food allergy, gluten intolerance (in the present study both are included in the food hypersensitivity concept), and diabetes are all diet-regulated conditions and have in common the fact that they all involve living in the presence of potential health problems, but not necessarily actual symptoms.

The important task for the interviewed food-hypersensitive adolescents in managing food hypersensitivity was to keep control of her/his food intake in order to avoid certain food. This never-ending work of constant vigilance and decision-making was not only described as the hard part of being food-hypersensitive, but also as time-consuming and frustrating. The adolescents experienced that the effort this work involved was underestimated and not fully understood by others. Muños-Furlong [22] has described in detail the extensive work with label reading, adapting recipes, selecting restaurants etc, that are all part of the daily coping strategies for families with a food-allergic child.

The food-hypersensitive adolescents' expression "to keep control" differs from how adolescents with diabetes describe a sense of "being controlled" by or "tethered" to their diabetes condition [21,23]. Both aspects of the control phenomenon are probably observable in both conditions. The category *Feeling constrained *can also to a certain extend be interpreted as containing aspects of a "being controlled"-experience, for example by limited choices of eating places, even if they are not characterised by the adolescents with the expression "being controlled". There is a difference in actions between the two conditions, which might explain the turn of phrases: the food-hypersensitive adolescent has to *avoid *unsafe foods, while the diabetic adolescent has to *supply *her/his body with either insulin or something to eat, depending on the blood-sugar level being high or low. The food-hypersensitive adolescents described that they had grown accustomed to being food-hypersensitive and this was now a matter of course. This way of identifying themselves with their food-hypersensitivity condition also differs from findings in diabetes research. The adolescent *is *food-hypersensitive but the diabetic adolescent *has *a disease [23].

There are several parallels between adolescents' experiences of being food-hypersensitive and adolescents' experiences of living with diabetes [21] as well as the experiences of adults with gluten intolerance [24,25], for example unwanted attention, worries about being a bother, and fear of complications/reactions. However, one finding from research on adults with gluten intolerance [24], which did not appear in the interviews with the food-hypersensitive adolescents, was the emotional shame. This emotion was described by the adults, but not mentioned by the adolescents. Instead, they described feelings of embarrassment, linked to the experiences of unwanted attention and perceptions of being a bother. Maybe this is just a reflection of different language usage in different age groups. However, the words may be a sign of different attitudes towards hypersensitivity – something shameful among adults, but in younger age something that puts you in embarrassing situations, for example when attracting attention by asking for safe food. This difference in attitudes might be related to how accustomed or adapted the individuals were to their food hypersensitivity. The usual time for the onset of the hypersensitivity condition was for the adults in adulthood and for the adolescents in early childhood. In comparison with the adults, the adolescents appeared to a larger extent to have incorporated the hypersensitivity into their lives and identity.

There is a phenomenon of disclosure avoidance reported for adolescents with diabetes [26,27] and this was also described by the food-hypersensitive adolescents in the present study. When they decided not to tell about their condition, this was done to avoid being troublesome to others and/or a way of avoiding unwanted attention and annoying questions. Disclosure avoidance has also been reported for adults with gluten intolerance, though other explanations were given. The adults felt so ashamed of their gluten intolerance that their avoidance could involve lies and cause negative consequences in social life [24].

The adolescents described experiences of unwanted attention. Such experiences are also described by adolescents with diabetes [21]. However, the food-hypersensitive adolescents also described themselves as courageous and persistent in coping with food avoidance in a satisfactory way. A strong sense of personal competence and ability to cope with their food hypersensitivity was emphasised. The adolescents said that they knew what they could eat and what they had to avoid. They knew how to check for unsafe foods and they requested safe food when it was needed. Moreover, as long as there was no risk of acute, life-threatening reactions, the food avoidance could be negotiated. Both in food hypersensitivity and diabetes [21] the adolescents could sometimes 'stretch the rules', and decide that an occasional intake of 'forbidden' food was worth the potential inconveniences.

However, according to a study from the United States, adolescence is the age group with most fatal reactions due to the intake of unsafe food [14], sometimes despite the comprehensive labelling of ingredients. Whether this kind of risky behaviour is a consequence of wide-awake risk-taking or a sense of invulnerability due to their youthfulness has been discussed [28,29]. Although adolescents know about the risks, and they have good risk-assessment knowledge, there are situations where they do "not think" at all – they just do [30]. This kind of behaviour is probably not possible to alter by education, because the adolescents already have the needed knowledge. To expose oneself to risks that could have been avoided, such as eating unsafe but correctly labelled food, might be a consequence of a "keeping-control collapse" rather than of risky decision-making. Perhaps there are ways of minimising the risk of this happening, for example by measures to lighten the burden of constant vigilance and decision-making rather than more education and demands on the individual adolescent to be unerring.

In their efforts to keep control the adolescents had to interact with people in their surroundings. Family, friends, and health-care providers were described as supportive and helpful. The home was the only place where the adolescents felt totally secure and confident that they were not at risk of getting something unsafe to eat. The family setting has been shown to be of significant importance in maintaining normality and ordinariness in families with a member diagnosed with gluten intolerance [31]. A couple of studies have actually shown improved family cohesion in families with a food-hypersensitive child [3,4].

Being far away from home involved increased feelings of insecurity and caused extra vigilance. This agrees with research showing that travels are an occasion when most persons carry their adrenalin pen with them. In daily activities, such as being with friends or doing sports, the adrenalin pen was often left behind at home [26].

To be food-hypersensitive was not considered by the adolescents themselves to be something extraordinary. Instead it was the incompetent and inadequate responses from others that were regarded as special. Friends of the same age were supportive, but adults questioned and forgot. This might be explained as being a consequence of a generation gap.

According to Sampson et al[26], the adolescents who are at risk of severe food reactions want their friends to be made aware of food allergy. The complaints about the ignorance of others, which the adolescents reported in the present study, indicate that they could be helped by such an intervention. At the same time, the adolescents say that they don't want to attract attention through their food hypersensitivity. The adolescents might have best use of a general increase in knowledge rather than direct educational measures about their specific food hypersensitivity as far as their classmates are concerned – which might be stigmatising instead of normalising.

### Methodological considerations and research implications

The adolescents in the present study were recruited from a sample of a previous study, which comprised food-hypersensitive children and adolescents with exclusion diets at school. Thus, the participants were considered to be at risk of adverse food reactions though they were not necessarily patients at a medical clinic. Since it is known that many individuals with food-hypersensitivity problems do not contact health care services about their hypersensitivity [32], this kind of sample makes it possible to get a broader picture of the food-hypersensitivity phenomenon than given by a sample recruited from health-care clinics.

There is a possibility that, at the time of the present study, some of the adolescents had outgrown their food hypersensitivity and were no longer at risk of adverse reactions. However, they perceived themselves as being food-hypersensitive and avoided foods that they regarded as unsafe. Their experiences of food avoidance can thus be considered as equally valid as those with persistent food hypersensitivity.

No comparisons are made between adolescents with different kinds of food hypersensitivity. One can presume that adolescents with life-threatening allergies have partly different experiences than adolescents with for example gluten intolerance. However, the aim of this study was not to differentiate between different kinds of food hypersensitivity, but to illuminate adolescents' experiences of being food-hypersensitive by presenting a "map" of different aspects of the food-hypersensitivity experience.

In qualitative research the findings are evaluated in terms of trustworthiness (credibility, dependability, and transferability) instead of the conventional criteria of validity and reliability [19].

The assessment of credibility involves the evaluation of two aspects: how the investigation was carried out and how the credibility is demonstrated [33]. Two interview techniques, focus-group interviews and individual interviews, were used. Both interview techniques have their advantages [34], and by combining them it was possible to include group discussions as well as individual statements about the food-hypersensitivity experience in the unit of analysis. The interviewed adolescents' characteristics showed a rich variation (Table 1) and thus contributed to a richer variation of the experience of being food-hypersensitive [19]. During the analysis process, the interpretations were compared and discussed by the authors until consensus was reached. The analytical concepts used in this study (Table 2) were presented to a reference group to test their trustworthiness. The reference group included two nurses and two psychologists with long clinical experience, and one sociologist – all highly knowledgeable about research methodology. Furthermore, credibility of the analysis was demonstrated at an open lecture for allergy nurses [19,33].

Dependability refers to the stability of data over time [19,33]. The data collection was extended over a period of six months. Due to insights into the food-hypersensitivity phenomenon during the interview process, the follow-up questions were developed [19]. However, all interviews were guided by a number of interview areas decided in advance so the consistency in the data collection, i.e. the dependability, is deemed good.

The descriptions of context, selection and characteristics of participants, data collection and process of analysis make the reader's assessment of transferability to other groups and contexts possible [19,33]. Because of the nature of the sample, the transferability of the findings in this study can be questioned. However, the characteristics of the interviewed adolescents showed a rich variation. The sample included boys and girls in a teenage range from 14 to 18 years. The offending food items as well as the types of adverse reactions reported showed a rich variation and included several common food-hypersensitivity patterns [13,35,36]. The onset of adverse food reaction varied between early childhood and mid-adolescence. There were adolescents with and without positive diagnostic tests [18] as well as adolescents with and without other family member(s) with food hypersensitivity [3] (Table 1). In these aspects, the sample can be seen as representative for food-hypersensitive adolescents in Sweden and thus the findings are transferable within a cultural context comparable to Sweden. Furthermore, transferability is enhanced by a comprehensive presentation of findings and by including quotations from the participants in the study findings [19].

The present study identifies adolescents' experiences of being food-hypersensitive. But to understand the mechanisms underlying the adolescents' experiences it is necessary to do in-depth studies of each identified category. Furthermore, the interrelationships between the categories need to be further explored. The role of socioeconomic factors and gender has not been investigated in the present study and therefore has to be taken in account in future studies of food-hypersensitivity experiences.

## Conclusion

This study adds to what is known about the impact of food hypersensitivity on individuals' everyday life by identifying a range of experiences reported by food-hypersensitive adolescents. The experiences of being food-hypersensitive should be considered in the context of adolescence as a developmental period – biological, cognitive, emotional, and social.

The adolescents' self-perceptions of being food-hypersensitive showed the presence of a food hypersensitivity identity of "being particular". This self-perception can be presumed to influence the adolescents' management of their food avoidance.

The way the food-hypersensitive adolescents described their experiences were somewhat contradictory. On the one hand they disclosed negative emotional reactions and obstructive experiences. On the other hand they express that it is "no big deal" to be food-hypersensitive. They revealed feelings of frustration, embarrassment, anger and sadness over being food-hypersensitive and what it brought along. But, they were also striving to normalise the condition and its consequences, their food avoidance and the responses of others. One important finding is that the adolescents experience ignorance of others with regards to their hypersensitivity condition.

### Clinical implications

The findings from this study have implications for all of those who deal with adolescents who are food-hypersensitive, and not only health professionals. Obviously, food avoidance by itself, and not only the somatic food reactions leads to consequences with significant impacts on adolescents' lives. A deeper insight into adolescents' experiences gives an understanding which can improve the care-givers' efforts. From a general perspective the findings point to a need for increased public awareness of food hypersensitivity and its consequences, for example in public areas such as restaurants and theatres. To lighten the adolescents' burden of keeping control, correct and distinct labelling of ingredients on food items in stores should be a matter of course. Also, better knowledge of the affected adolescents' experiences might help those around the individual, such as friends and school personnel, to be supportive in a constructive way.

## Competing interests

The author(s) declare that they have no competing interests.

## Authors' contributions

BM conceived of the study and carried out the acquisition of data. BM, GN and BW were involved in the data analysis. BM drafted the manuscript. GN, BW and SA have been involved in revising it critically for important intellectual content. All authors read and approved the final manuscript.

## Pre-publication history

The pre-publication history for this paper can be accessed here:


